# Dynamic Light Scattering Spectroscopy as an Early Biomarker of Alzheimer’s Disease

**DOI:** 10.1002/alz.083932

**Published:** 2025-01-09

**Authors:** Jeffrey Neill Weiss

**Affiliations:** ^1^ Parkland, FL USA

## Abstract

**Background:**

The early detection of neurologic damage at the microscopic level when the disease is subclinical would facilitate intervention preventing progression or potentially reversing the condition. The early determination of drug efficacy could shorten the length of drug studies, thereby reducing research costs. The eye is the only place in the body where an artery, vein, and nerve can be directly visualized The nerve fiber layer of the retina is an outgrowth of the brain.

**Method:**

Dynamic Light Scattering (DLS) Spectroscopy measures the thermal random movement (Brownian Motion) of particles by analyzing the temporal fluctuations of scattered light.

A proof‐of‐concept instrument for making retinal measurements provides optical power well below the maximal permissible exposure recommended by the ANSI Z136.1 standard. Scattered light is analyzed by a digital autocorrelator with an extended delay option for baseline determination. The testing duration is 5 seconds.

DLS measurements were made from the macular area of 4 patients diagnosed with mild cognitive impairment and 4 age‐matched controls with no history of neurologic disease. Both groups of patients had normal retinal examinations, including normal Optical Coherence Tomography, (OCT).

**Result:**

Figure 1 Screenshot ‐ 62‐year‐old female recently with mild cognitive impairment, subsequently diagnosed with Alzheimer’s disease 1 year after DLS testing.

Figure 2 Screenshot ‐ Age‐matched control

**Conclusion:**

The 4 patients with mild cognitive impairment were subsequently diagnosed with Alzheimer’s disease approximately 1 year after the abnormal DLS measurement. The differences between the patients subsequently diagnosed with Alzheimer’s disease, compared to the normal age‐matched controls, was consistent across the tested patients. Alzheimer’s measurements begin above 0.6 g 2(t)‐1 and exhibit flattening of the initial line as opposed to the curve seen in non‐Alzheimer’s patients.

The development of an early, noninvasive, quantitative test to diagnose Alzheimer’s disease will lead to breakthroughs in drug development and hopefully, to the successful treatment of patients before dementia is irreversible.

